# Effect of TiO_2_ nanoparticles on the tribological properties of lubricating oil: an experimental investigation

**DOI:** 10.1038/s41598-022-09245-2

**Published:** 2022-03-25

**Authors:** Corina Birleanu, Marius Pustan, Mircea Cioaza, Andreia Molea, Florin Popa, Glad Contiu

**Affiliations:** 1 MicroNano Systems Laboratory, Technical University from Cluj-Napoca, Blv. Muncii nr. 103-105, 400641 Cluj-Napoca, Romania; 2 Department of Automotive Engineering and Transports, Technical University from Cluj-Napoca, Cluj-Napoca, Romania; 3 Department of Materials Science and Engineering, Technical University from Cluj-Napoca, Cluj-Napoca, Romania; 4 Depatment of Manufacturing Engineering, Technical University from Cluj-Napoca, Cluj-Napoca, Romania

**Keywords:** Engineering, Mechanical engineering

## Abstract

Nano-lubricants offer improved tribological properties in many applications, such as machines and engines. The presence of nanoparticles in the lubricating oil affects its wear, friction, thermal, chemical and physical properties in many ways. Titanium dioxide (TiO_2_) is a promising lubricant additive for enhanced engine efficiency. This article reports the effect of 10 W-30 pure base engine oil suspended TiO_2_ nanoparticles. Four different volume concentrations (0.01%, 0.025%, 0.050% and 0.075%) of TiO_2_ nanoparticles in the base lubricating oil are used for the analysis. The tribological tests were performed at ambient temperature as well as at 75 °C using a four ball tribometer. Scanning electron microscope (SEM) and Alicona Inginite Focus G5 microscope were used to analyze the worn surface. The results show that the surface-modified TiO_2_ nanoparticles can remarkably improve the load-carrying capacity, the friction reducing, and anti-wear abilities of the additive oil. The diameter of the wear trace and the coefficient of friction are the tribological properties analyzed for the nano-lubricant prepared at different volume concentration (VC). It was found that the diameter of the wear scar and the coefficient of friction increase with increasing VC of TiO_2_ nanoparticles in the lubricating oil. The main objective of the paper is to present the recent progress and, consequently, to develop a comprehensive understanding of the tribological behavior of engine oil mixed with TiO_2_ nanoparticles.

## Introduction

Research conducted in recent years on the addition of nanoparticles (NPs) to oils used in industry by increasing the load-bearing capacity of friction parts in mechanical systems highlighted the friction modifiers and improved the properties of anti-wear. Moreover, increasing the severity of loading and speed conditions in machines is a constant challenge for tribologists to develop improved solutions to increase the performance properties of used oil. Nanoparticles, due to their small size, can access areas with extremely small surface roughness and therefore have great potential in improving the tribological properties of lubricants and contact surfaces. Numerous studies have been conducted in the last two decades regarding the use of nanoparticles as lubricating additives^1–3^. The addition of nanoparticles to the base oil can reduce friction and wear, and it can be said that the nanoparticles could be beneficial lubricating additives, although some may be hard and abrasive^4–7^. In recent years, various nanoparticles have been investigated^4,6–8^. The nanoparticles used are generally metals such as Cu, Ni, Mo, Ag, and Pd; metal oxides including TiO_2_, SiO_2_, ZnO, ZrO_2_, and CuO; and sulfides WS_2_, MoS_2_, and PbS^2^.

Several hypotheses emerge from the open literature about how nanoparticles contribute to the reduction of friction and wear under different laboratory testing conditions. However, useful information can be detached if prototypical materials are used together with a balance between the applied mechanical parameters (loads, speeds, temperatures, contact pressures) and surface conditions.

The transfer and adhesion of the nanoparticles leads to the change of the surface condition, the self-reduction, and the formation of a thin TiO_2_ tribo-film which conduct to the decrease of the coefficient of friction, of the pressure and the temperature in the contact area, therefore of the wear phenomenon.

The addition of TiO_2_ nanoparticles to the lubricating oil showed stable friction due to the formation of protective films on the worn surfaces^9^. Shenoy et al.^10^ analyzed the influence of TiO_2_ nanoparticles in lubricating oil. The results obtained showed a higher bearing capacity by approximately 35% compared to the use of lubricating oil without the addition of nanoparticles. The experiments performed by Kao and Lin^11^ using an alternative sliding tester to analyses friction and wear in the presence of additive rapeseed oil showed that there was an 80.84% reduction in mean surface roughness. The average diameter of TiO_2_ was 50 nm, and the particle concentration was 5 weight percent (wt.%). Using a low concentration of TiO_2_ nanoparticles is enough to improve the tribological characteristics. The coefficient of friction and the wear scars decreased by approximately 15.2% and 11%, respectively^12^.

Several studies have been conducted on nanoparticles as oil additives^10–17^. The particle size of TiO_2_ affected the wear behavior of the composite material^18^. It was found that the microscale particles of TiO_2_ damaged the surface due to severe adhesion and abrasion. The surface damage due to nanoscale TiO_2_ particles was due to slight abrasion. More investigations have been carried out on TiO_2_ as a coating material^12,13,18–21^ or reinforcement in composites^10^ for better tribological performance^13^. Gupta et al.^14^ presents for some commercial lubricants the friction coefficient (COF) values in different experimental conditions for instance: for SAE20W40 the µ = 0.102 under testing parameters: 200 rpm, 392 N, 75 °C, 3600 s, for mineral oil µ = 0.104 under testing parameters 20 Hz, 10 N, 3600 s. Anand et al.^20^ studies the effects of varying concentrations by weight (0.5 and 1 wt.%) of rice bran oil/TiO_2_ nanofluid an revealed an improvement in the viscosity with an increase in nanoparticle concentration. Furthermore, an improvement in viscosity in hybrid fluids at higher temperatures was observed.

**Figure 1:**
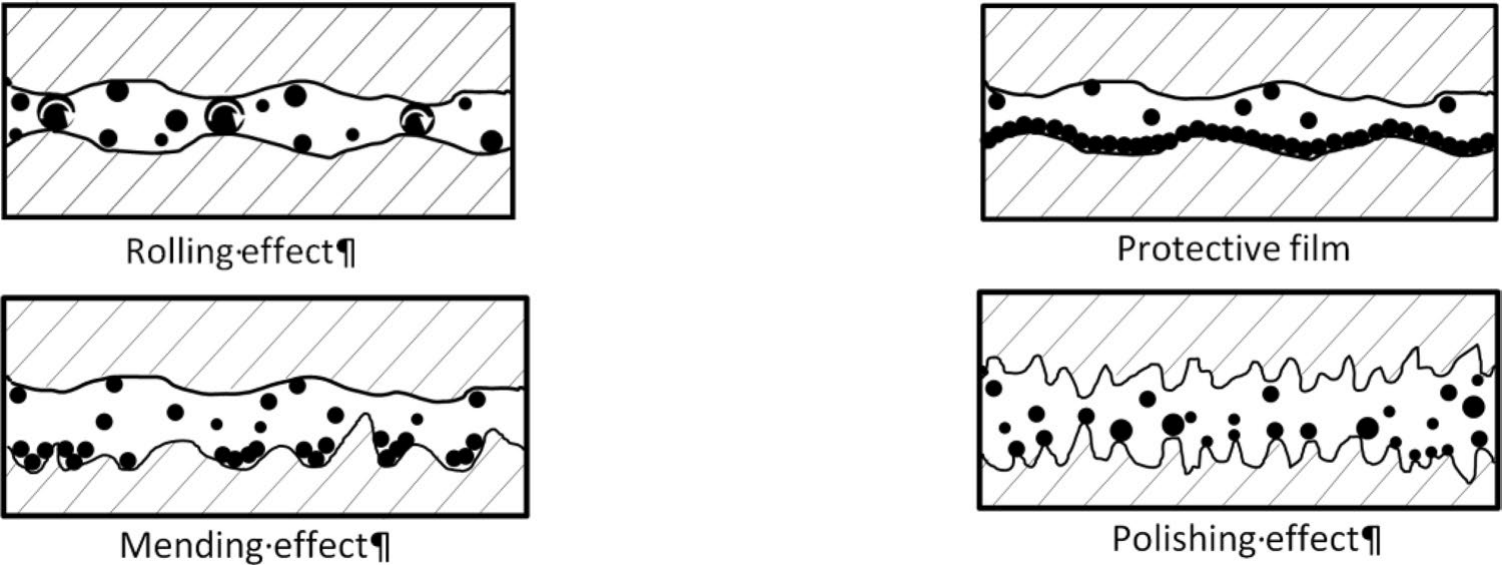
Lubricating mechanisms by NPs based lubricant^5,15^.

The lubrication mechanism in the presence of nanoparticles produces (Fig. 1): (a) the surface properties will be modified and two friction surfaces will be separated with tribo-film formation, thus offering promising tribological performance; (b) the nanoparticles roll between the friction surfaces, reducing friction and wear; and (c) the heat and pressure generated during operation lead to the compaction of the nanoparticles following wear, which is considered a mending effect of the surface and a polishing effect^7^.

In this paper, the antifriction and antiwear behaviors of TiO_2_ nanoparticle suspensions in 10 W-30 oil with different percentages (0.010, 0.025, 0.050 and 0.075 wt.%/v) were evaluated using a tribometer with four balls. The results present the influence of the nanoparticle percentage on the tribological behavior of the mixed oil, the evaluation of worm surface structure through the various operating conditions tested by scanning electron microscopy (SEM), and the depth of wear marks on the spheres measured using the Alicona Inginite Focus G5 Microscope.

A 4-ball tribometer was used in this study, for which the friction force (the coefficient of friction) between the samples was continuously recorded according to the normal load and the elapsed time. Four nano-oil samples were prepared and tested repeatedly with various specimens from the 4-ball tribometer to evaluate the direct effect and the surface-enhancing effect of nanoparticles in the lubricating oil.

Therefore, the purpose of this paper is dedicated to finding the possibility of tribological performance improvement of conventional engine oil using TiO_2_ nanoparticle additives. This promising technology has a large impact on fuel consumption and engine durability for a greener future.

## Samples description

Experimental studies involve the formulation of stable TiO_2_-based nano lubricant samples at different concentrations. The physicochemical properties, such as density, viscosity, and viscosity index, were measured using an automated SVM 3000 Anton-Paar rotational Stabinger viscometer. Density was measured using a vibrating U-tube densimeter, and for the determination of viscosity, a Peltier element was used to thermoregulate the samples.

### Materials

Against the background that TiO_2_ is best suited for many tribological applications (including solid lubricants) due to its excellent tribological behavior, these nanoparticles were chosen for the current investigation. Commercial TiO_2_ Degussa P25 nanoparticles supplied from Sigma–Aldrich and Motul 5100 4T 10 W-30 engine oil were used in the formulation of nano lubricant samples. The average sizes of the TiO_2_ nanoparticles were 18–21 nm.

The properties of the raw materials are presented in Table 1. The varying concentrations of TiO_2_ for the engine oil used in the experiments were 0.010, 0.025, 0.050 and 0.075 wt.%, as shown in Table 2. To demonstrate the effect of nanoparticles on the engine oil additives, tribological tests were conducted using commercial lubricant engine oil—Motul 5100 4T10 W-30.

**Table 1:** Properties of commercial TiO_2_ Degussa P25 and Motul 5100 4T 10 W-30 engine oil.

Raw materials	Properties	Value
TiO_2_ Degussa P25	Crystalline phases	75% anatase, 25% rutile
	Average particle size	21 nm
	Surface area	> 30 m^2^/g
	Density	3900 kg/m^3^
Motul 5100 4T	SAE Viscosity grade	10 W-30
	Density at 20 °C	871 kg/m^3^
	Viscosity at 40 °C	74.6 mm^2^/s
	Viscosity at 100 °C	11.5 mm^2^/s
	Pour point	− 36 °C
	Flash point	226 °C
	Total basicity number (TBN)	7.5 mg KOH/g

**Table 2:** Concentrations of the prepared nano lubricants.

Lubricant sample	LS	L0	L1	L2	L3
TiO_2_ (wt.%)	0	0.010	0.025	0.050	0.075
Pure base oil (%)	100	99.99	99.75	99.50	99.25

### Nano lubricants preparation

The mixing of nanoparticles with engine oil is an important step towards the improvement of engine oil quality. Different amounts of TiO_2_ nanoparticles (Table 2) were dispersed into engine oil, and 0.2 mM Triton X surfactant was added to increase the stability of the nano lubricants. The 0.2 mM Triton X surfactant plays an important role in blending the nanoparticles in a manner that makes them soluble in the engine oil, also providing stability for the nanoparticles, preventing the agglomeration of the nanoparticles within engine oils. The uniform dispersion of TiO_2_ nanoparticles in base oil is a challenge because of the high surface energy of the nanoparticles, they tend to agglomerate and settle down^13^. Hence, before friction tests, to increase the stability over time, the prepared suspensions were subjected to magnetic stirring, followed by ultrasound treatment for 30 min. For preparation of the nano lubricants was used both mechanical and chemical methods to increase the stability. Thus, in the first stage, 0.2 mM Triton X surfactant was added in the suspensions and in the second stage, the magnetically stirring, followed by an ultrasound treatment were used. These methods were used to decrease the agglomeration effect which acquired when the Brownian motion and Van der Waals intermolecular attractive forces from the suspensions were greater than the repulsive forces^16,22^.

**Figure 2:**
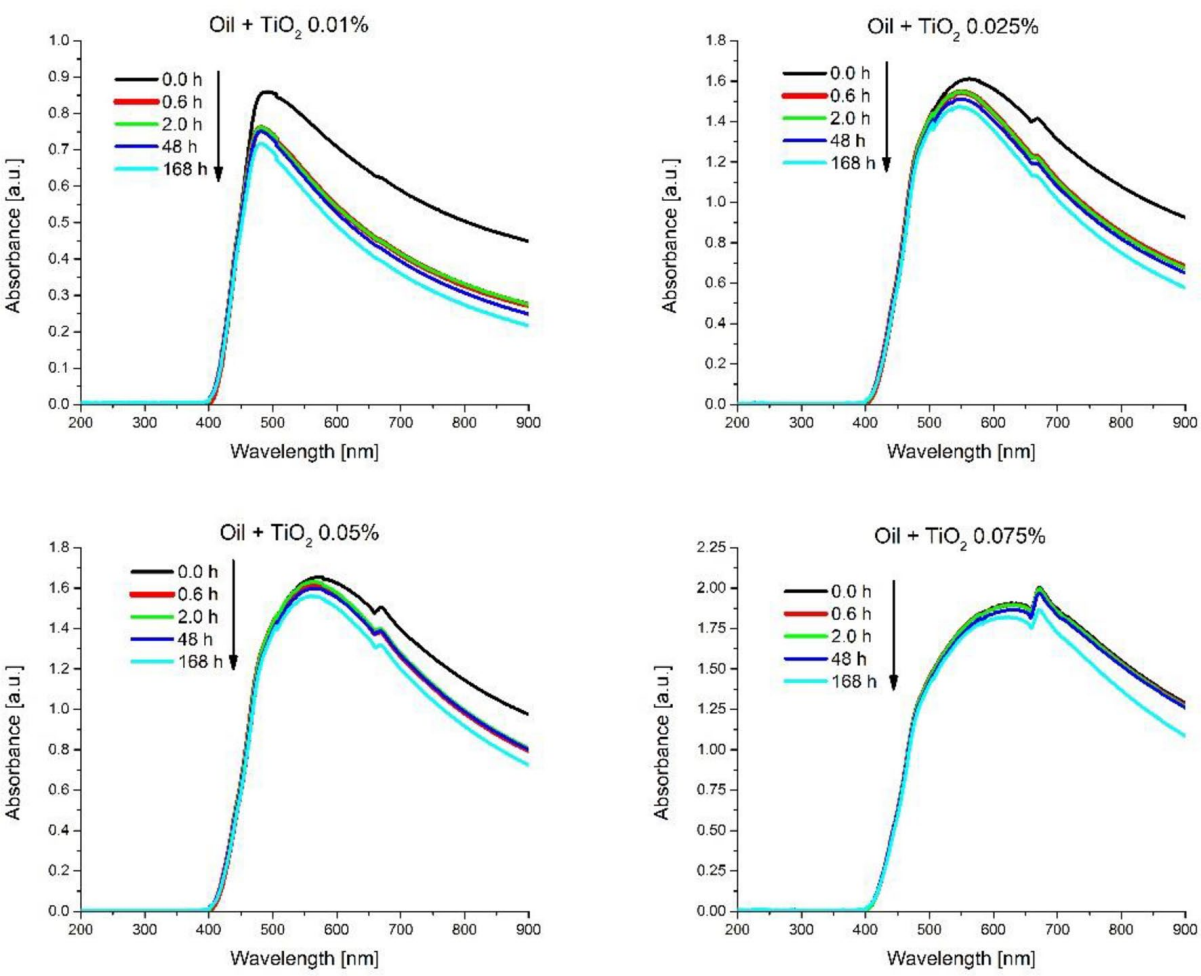
UV–Vis absorption spectra of the TiO_2_ nano lubricants in time.

The stability studies were performed using UV–Vis JASCO V-550 spectrometer, with scanning rage between 200–900 nm and the baseline was made with oil lubricant and surfactant mixture due to emphasizing the absorption band of TiO_2_ (monitoring of TiO_2_ concentration from the suspensions). Thus, in Fig. 2 are presented the UV–Vis spectra of the TiO_2_ nano lubricants. A decrease of the UV–Vis absorbance was observed after 2 h for the nano lubricants, while at 168 h it was observed that the absorbance tends to maintain at the same value. This is attributed to the coating of the nanoparticles with the surfactant molecules, which due to the large molecular chain; separate the nanoparticles from each other, reducing the agglomeration effect, a phenomenon called electrostatic stabilization^23^.

The physicochemical properties of engine oil and TiO_2_ nano lubricants are presented in Table 3. An extended discussion regarding the physicochemical properties of TiO_2_ nano lubricants were presented in previous work^24^. It can be observed that with an increase in the TiO_2_ amount in the engine oil, the density tends to increase by 0.2% (from 862 kg/m^3^ for the base oil up to 864 kg/m^3^ for the nano lubricant, which contains 0.075 wt.%/v) due to the high density of the nanoparticles (3900 kg/m^3^), even if the concentration of TiO_2_ is very small. Additionally, kinematic viscosity at both 40 °C and 100 °C tends to increase with TiO_2_ concentration in nano lubricants to 3% and 4%, respectively (from 89.7 to 92.3 mm^2^/s and from 13.6 to 14.2 mm^2^/s, respectively). According to Ali et al.^16^, this behavior is related to the fact that the nanoparticles act as catalysts in a cracking reaction and have quite good heat transfer properties. The high viscosity of the nano lubricant oils improves the lubricating property because it reduces friction and prevents rapid wear. The viscosity index increases up to 158 when TiO_2_ nanoparticles are added into the oil, which means that the variation of viscosity with temperature is lower for nano lubricant oils than base oil.

**Table 3:** The physicochemical properties of engine oil and TiO_2_ nanolubricants.

Sample	Density at 15 °C (kg/m^3^)	Kinematic viscosity at 40 °C (mm^2^/s)	Kinematic viscosity at 100 °C (mm^2^/s)	Viscosity index
LS (pure base oil)	862	89.7	13.6	154
L0	863	91.6	14.1	158
L1	863	92.1	14.1	158
L2	863	92.0	14.1	158
L3	864	92.3	14.2	158

### Ball materials

The standard ball test material was 52100 grade 25 chrome alloy steel, 12.7 mm in diameter with a surface roughness (R_a_) of 0.1 μm, extra polish (EP) grade of 25 and hardness of 54–58 HRC. Four new balls were used for each test. Before starting a new test each time, the balls were cleaned with a technical cleaner (isoparaffinic-based solvent cleaner) and wiped dry using tissues. 52100 steel is commonly used as ball and roller bearings in industry and for a variety of automotive applications. It is known for its excellent surface quality, superior wear resistance, hardness, and high load capacity. Some of the chemical composition and mechanical properties of 52100 steel used in this investigation are listed in Table 4.

**Table 4:** Chemical and mechanical properties of ball material.

Chemical properties						
%Fe	%C	%Si	%Cr	%P	%S	%Mn
96.5–97.3	0.93–1.1	0.15–0.35	0.14–0.16	0–0.025	0–0.015	0.25–0.45

Mechanical properties						
Hardness (HRC)	Tensile strength (MPa)	Yield strength (MPa)	Young's modulus (GPa)	Poisson’s ratio (–)	Roughness R_a_ (µm)	
54–58	2100–2200	2000	200	0.3	0.1	

## Experimental method and device

The American Society of Lubrication Engineers has published a catalog of friction and wears testing devices that describes in detail over 230 different tribometers^7^. Each device has its strengths and weaknesses. For this work, we used the four-ball wear test geometry, which has been selected to provide the following test conditions: high contact pressures that ensure operation in boundary lubrication mode; good control of the operating parameters of load, temperature, speed, operating time, atmosphere; high sensitivity of wear and friction measurements; simple samples for testing, small dimensions, and easy manufacture.

The four-ball tester is an excellent tool for checking and determining the quality of lubricants and additives. This tribometer can be used to determine the wear preventive properties (WPs), extreme pressure properties (EPs) and friction behavior of lubricants. The widespread acceptance of Four Ball Tester test results makes it an excellent tribological device.

**Figure 3:**
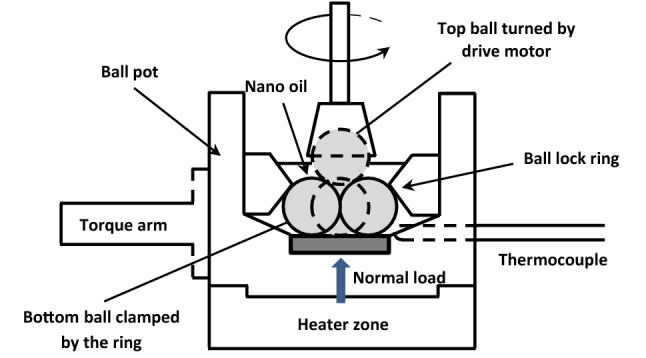
Schematic representation of four-ball tribometer.

**Figure 4:**
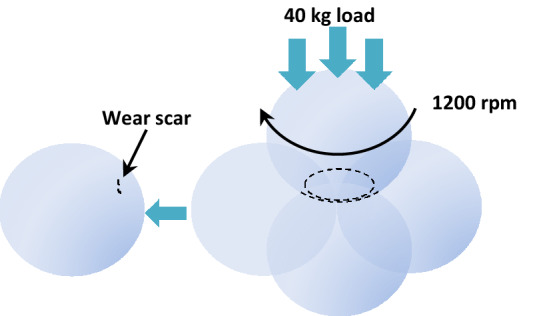
Four ball of tribological system.

This tribometer consists of a device where an upper ball can be rotated and is in contact with three fixed balls that are immersed in the oil sample. Different loads are applied to the ball by weights applied to a system with the load lever. The upper rotary ball is held in a special chuck located at the lower end of the vertical axis of an electric motor and rotates at a constant speed. The lower fixed balls are held in contact with each other in a steel pot by a clamping ring and a locking nut. The arrangement is illustrated in Fig. 3. The basic sample configuration (Fig. 4) consists of a tetrahedral arrangement of four balls.

These tests were carried out under the American Society for Testing and Materials (ASTM) condition and ASTM D 4172 method test B. The tests were conducted under dry conditions at a room temperature RT (23 ± 2)^o^C and then at (75 ± 2)^o^C and relative humidity RH (35 ± 2)%, the speed (1200 ± 60) rpm, the time test (30 ± 1) minutes and the load: (396 ± 4) N. The tests were repeated at least three times for every measurement. All presented results in this work are the arithmetic mean value of the measured values, unless otherwise stated. The corresponding standard deviation is indicated by the error bars, describing the scatter of the results.

**Figure 5:**
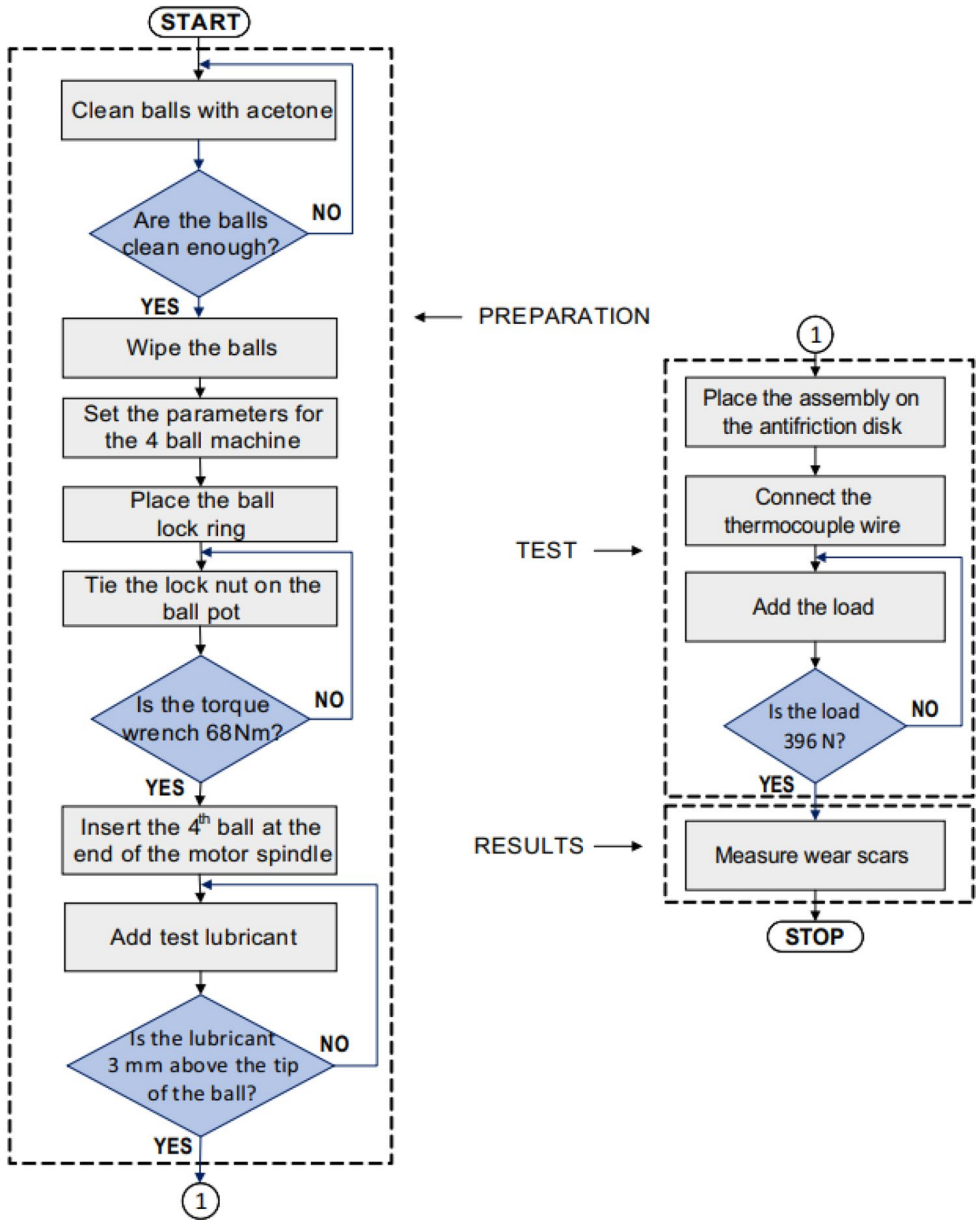
Four-ball tribometer—flow chart experimental procedure.

The experimental procedure can be followed in the flow chart shown in the Fig. 5.

The viscosity is a very important parameter for a lubricant, as it affects the film thickness and the wear rate of the sliding surface. It is used for the identification of individual grades of oil and for monitoring the changes occurring in the oil while in service. Increasing the viscosity normally shows that the used oil has been deteriorated by contamination or oxidation. Additionally, decreased viscosity usually indicates dilution of the oil. The oil viscosity was measured with a viscosity meter at experimental temperatures of 23 °C and 75 °C. In test B method ASTM D 4172, the load was 396 N, and in the four-ball test machine, the load cell was mounted 80 mm from the center of the shaft. In addition, the wear was measured with the average of horizontal and vertical scars with SEM, and the depth of wear marks on the spheres was measured using an Alicona Inginite Focus G5 microscope. The duration of the test was selected to ensure that the running-in period was less than 30% of the total duration of the test.

The RDS (Energy-dispersive X-ray spectroscopy) analysis of the samples before and after tribological tests was measured using Oxford EDS—Ultim^®^ Max EDS with AZtecLive software. AZtecLive enables the quick and comprehensive investigation of a sample with real-time chemical feedback via the AZtecLive interface.

## Results and discussion

### Wear scar images of balls

**Figure 6:**
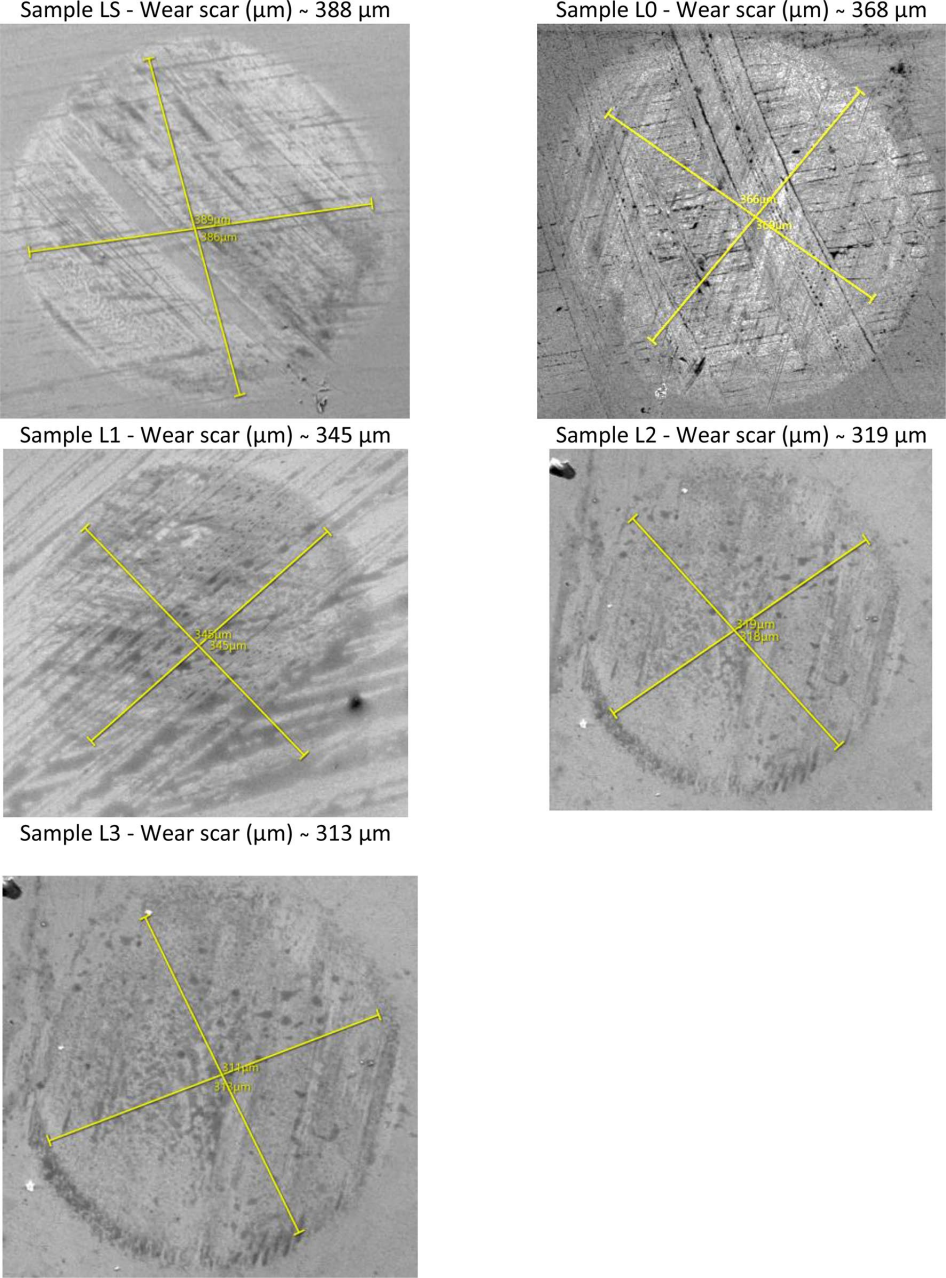
SEM images of worn-out samples corresponding to pure base oil and additive 0.01, 0.25, 0.050 and 0.075 wt.% TiO_2_ at RT, 396 N load at 1200 rpm after a 30-min period of testing.

**Figure 7:**
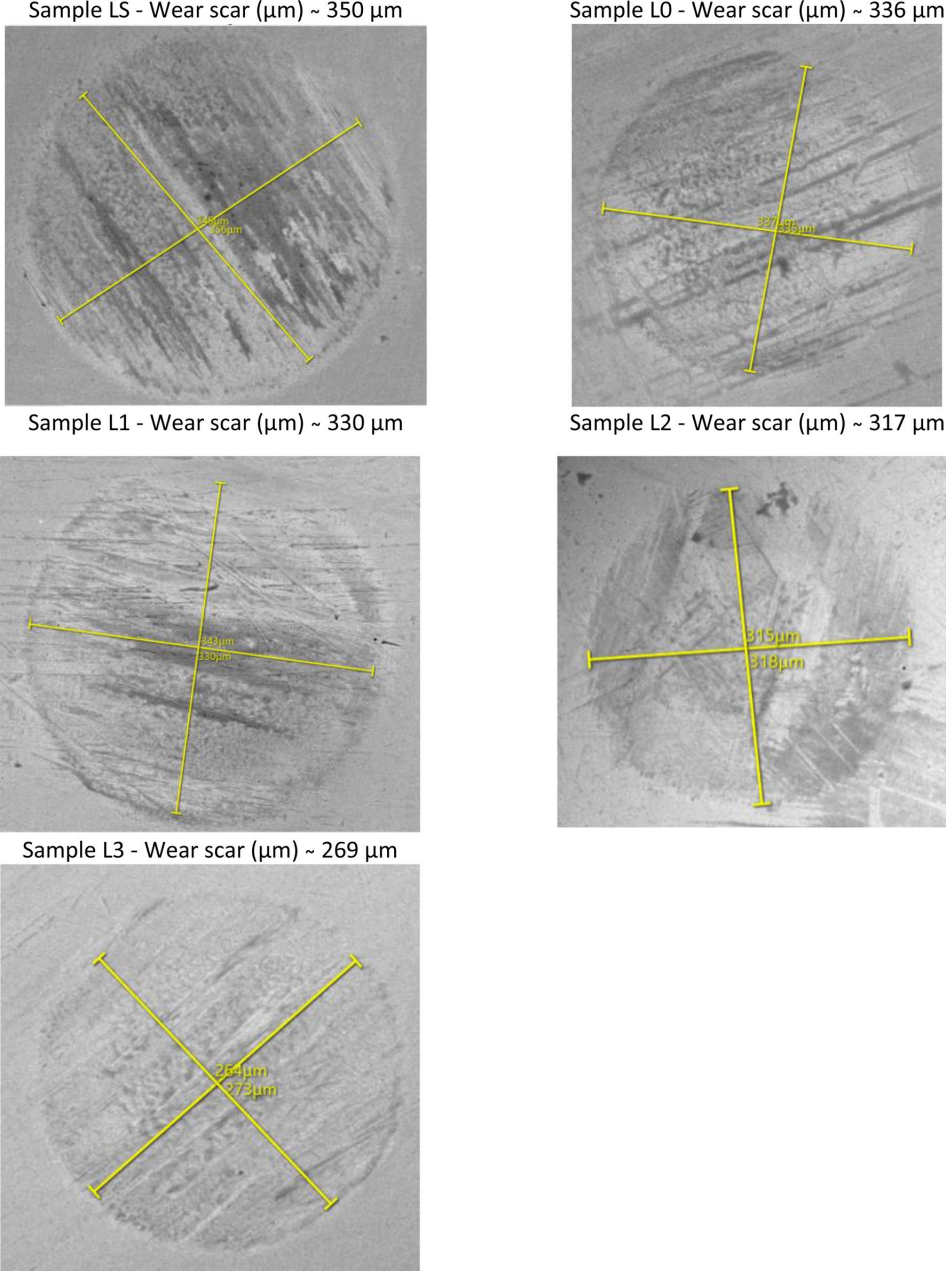
SEM images of worn-out samples corresponding to pure base oil and additive 0.01, 0,025, 0.050 and 0.075 wt.% TiO_2_ at 75 °C, 396 N load at 1200 rpm after a 30-min period of testing.

Micrographs of the wear scar formed on the balls for each of the formulations are shown in Figs. 6 and 7. The addition of TiO_2_ led to reduction in the diameter of the wear scar.

Figures 6 and 7 reveal the wear scar maps of the tested ball sliding against the bearing steel balls. This shows that the wear degree of the samples containing TiO_2_ was obviously improved compared to that of the base oil. When the base oil was a lubricant, the worn surface of the ball presented black sediment, which confirmed that the main component was Fe resulting from ball-on ball milling. Moreover, the 0.01 wt.% and 0.025 wt.% samples also presented back sediment, but the wear radii were visibly reduced. In particular, the radius of the 0.075 wt.% oil sample in the stabilized state after rubbing was smaller than that of the other samples, showing the superior anti-wear performance of this sample.

To further investigate the wear mechanism, SEM images of worn surfaces of the lower balls lubricated by the base engine oil and the engine oil samples containing 0.01, 0.025, 0.050 and 0.075 wt.% TiO_2_ nanoparticles were investigated. The tests were performed at two different working temperatures, 23 °C (RT) and with oil heated to 75 °C.

Following the evaluation of the wear traces with SEM, radius differences of the circular wear trace are observed, and the radius difference is approximately 5–10%. This should not greatly influence the mechanism of wear (generation of wear residues, increase of the tribo-layer, etc.).

Figure 6 shows that for samples LS and L0, there were obvious plowing and some pits on the worn track, which were due to the second local rupture of debris during the sliding process; moreover, the friction of debris at the sliding interface clearly furrows on the worn surface.

Figure 6 (L2 and L3) shows that the worn surface presented finer mesh-like grooves, in agreement with the COF result in Fig. 14. For the 0.05 wt.% sample, the worn surface was covered by fine grooves and detachments after the rolling test, as shown in Fig. 4 (L1), and the mechanism of wear was dominated by microplowing. Furthermore, Fig. 6 (L2 and L3) shows that the wear scratch was almost invisible; through a local zoom of the images in Fig. 6, slight furrows of wear could be observed. This is mostly attributed to the increase in TiO_2,_ which could reduce the wear of frictional interfaces. This indicates that TiO_2_ played a lubricating role and prevented wear in the rolling process. The above results agree with the tribological results showing the decrease in the friction coefficient in Fig. 14.

The wear scar diameter of each of the three bottom test balls was measured to determine the lubricity performance of the test lubricant. In general, the larger the wear scar diameter is, the more severe the wear, but we also consider the depth of the wear trace. The wear scar diameter was determined for each of the three fixed balls.

The temperature of the test lubricants was measured by a thermocouple attached to the four-ball tester to record the temperature changes throughout the duration of the experiment.

The base oil and nano-oils were tested at temperatures of 23 °C and 75 °C (close to the temperature service for engine oil). Increasing the temperature results in nanoparticle movement, which is associated with reducing the fluid resistance over the flow; therefore, the viscosity is reduced. With respect to viscosity, either of the base oils or nano-oils are non-Newtonian fluids. However, the temperature of the contact point for the balls is also influenced by the sliding speed. A sliding speed of 0.80 ms^−1^ (calculated based on input parameters) was selected to provide the minimum and extreme heating due to sliding.

Figure 6 provides SEM images of the rubbed surface lubricated with pure oil and TiO_2_ nanoparticles added with 0.01 wt.%, 0.025 wt.%, 0.050 wt.% and 0.075 wt.%, respectively at RT (room temperature). The worn surface lubricated with pure oil shown in Fig. 4—LS is rough with many thick and deep furrows due to the strong adhesion and ploughing between contacted asperities on the rubbed surface of the tribological pair. The worn surface of the ball when the base oil was used as a lubricant showed a combination of abrasive wear and adhesive. The surface damage was highlighted in these tests, which can be seen in Fig. 4—LS. The presence of oil at the interface reduced COF, but it fluctuated during the test. The hard debris generated during slipping in the oil could have further damaged the surface. The contamination with these particles (the third body) could have caused the COF fluctuation. However, there was no evidence of free debris on worn surfaces after these tests. The debris was not welded or formed a transfer film on the sliding surfaces.

The worn surface is lubricated with TiO_2_ nanoparticles added with oil is smoother than that lubricated with pure oil. And with the increase of the addition the concentration of TiO_2_ nanoparticles in pure, thick oil and the deep furrows on the surfaces of the wear scars become smaller and shallower. There was no indication that the nanoparticles had settled on the sliding surface. The deterioration of the surface was due to the combination of abrasive wear and adhesive.

TiO_2_ nanoparticles were deposited on the surface, acted as third body particles which further decreased the shear stress. These deposited nanoparticles were more stable than the debris of the sliding materials. It appears that the surface of the wear path has been smoothed. But there were some places where serious damage occurred. It is evident that the higher wt.% TiO_2_ content has higher polishing effect.

With the increase of the temperature (Fig. 7) it is seen that the surface of the wear track showed signs of wear that indicate wear of the adhesive with very slight abrasion marks. There was no indication of debris left on the surface. As the temperature rises, the addition of TiO_2_ nanoparticles to the base oil has less effect on wear. Usually, the surfaces have been worn due to the adhesive wear. However, the severity was different. It can be seen from Fig. 7 that the signs of worn surface area observed decreased with the increase in TiO_2_ wt.%. When the TiO_2_ nanoparticles were added to the base oil, the wear of the surface was due to micro abrasion and minimally due to the adhesive wear. The surface damaged by nanoscale TiO_2_ particles was caused by slight abrasion^13^. This observation is consistent with the current investigation. In addition, the presence of lubricant at the sliding interface could reduce the severity of abrasion due to nanoparticles.

### RDS analyses of the balls

The chemical characterization/elemental analysis of materials of the samples used in this investigation was done based on the EDS analysis using Oxford EDS—Ultim^®^ Max EDS with AZtecLive software. From the multiple set of test samples, several representatives were chosen for which this analysis was done.

**Figure 8:**
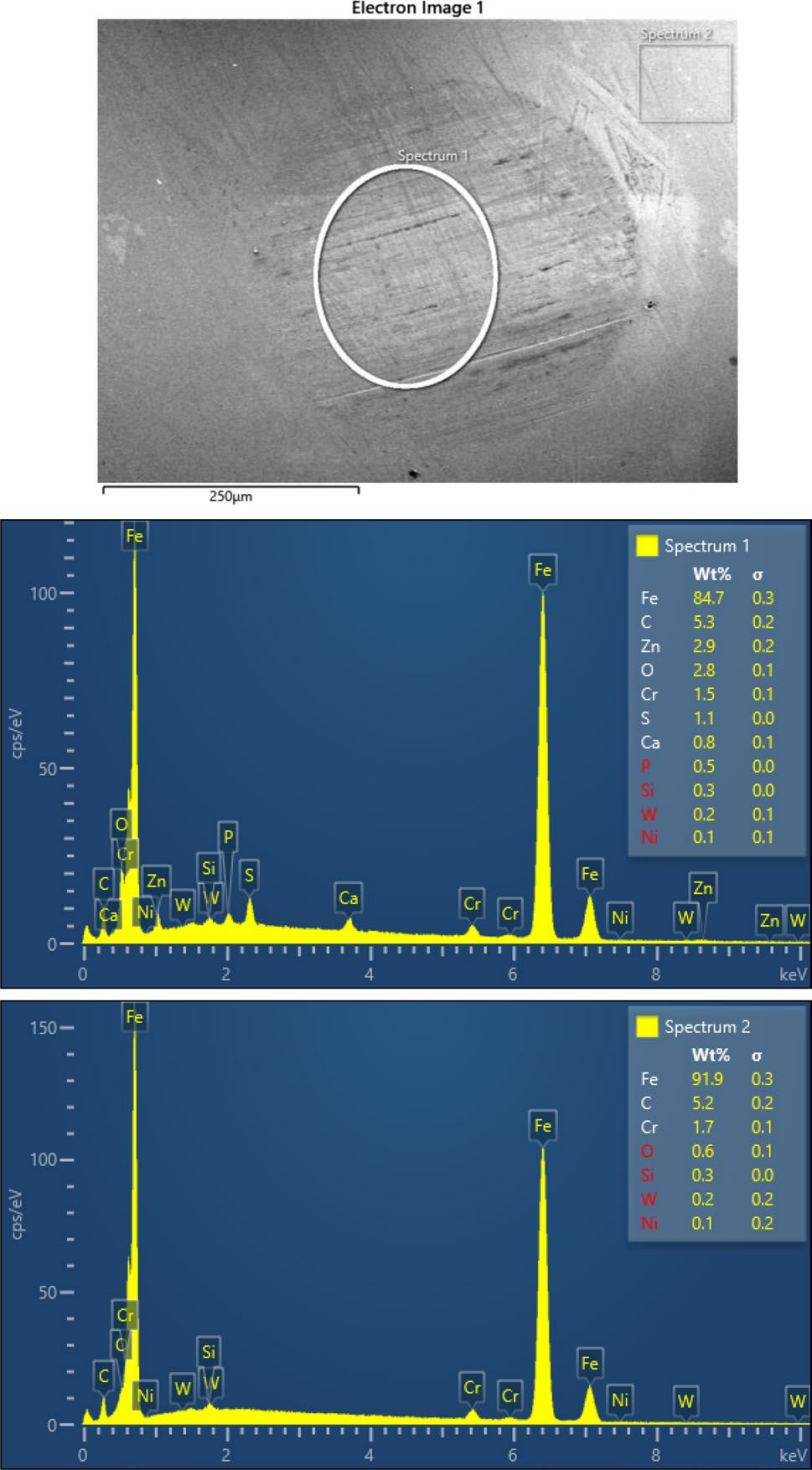
SEM and EDS analysis of worn surface of steel ball after four-ball test lubricated by the pure based oil at 75 °C.

**Figure 9:**
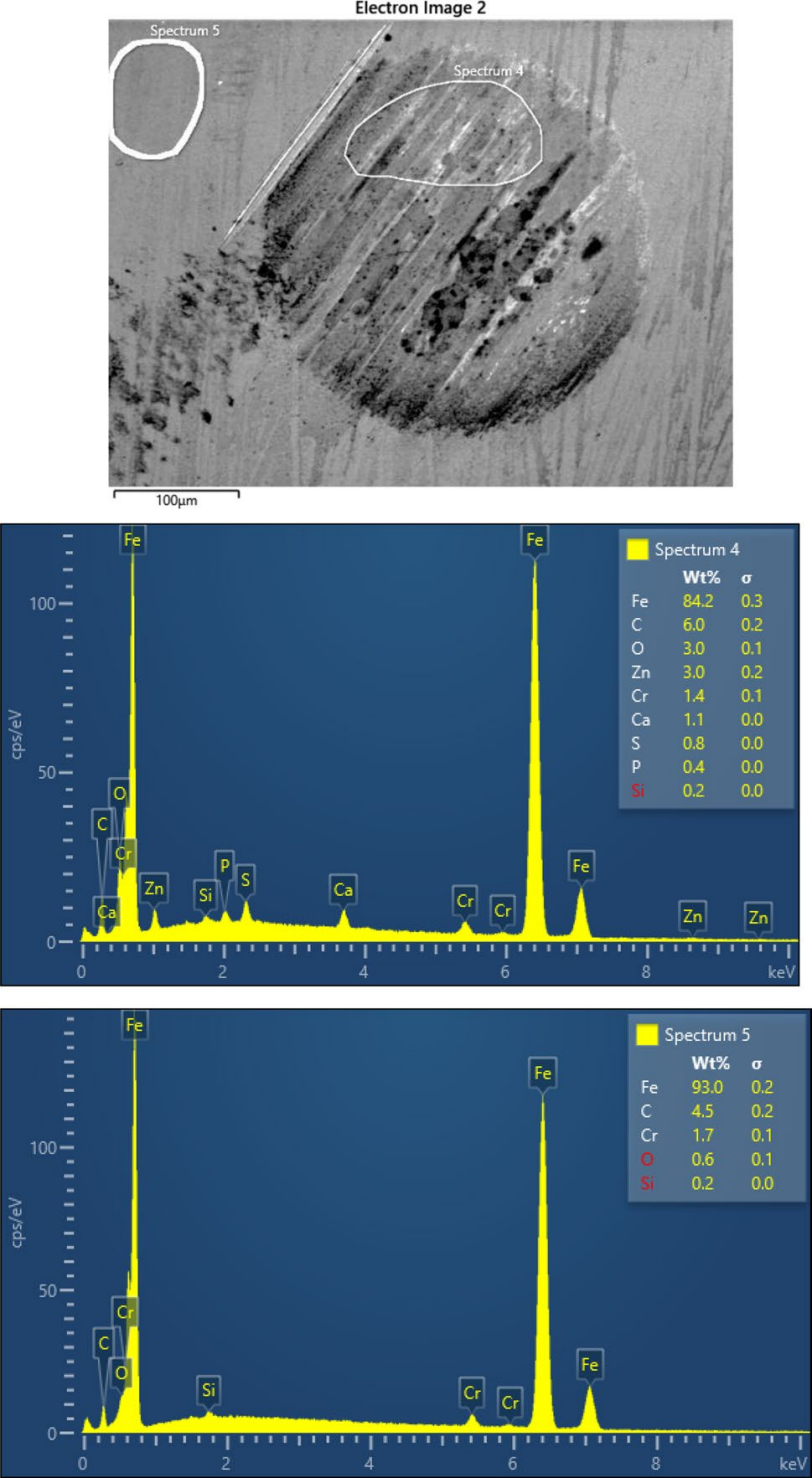
SEM and EDS analysis of worn surface of steel ball after four-ball test lubricated by the oil-added 0.075 wt.% TiO_2_ nanoparticles at 75 °C.

Figures 8 and 9 show the EDS images obtained for a 75 °C test with pure base oil and 75 °C test with the oil-added 0.075 wt.% TiO_2_ nanoparticles, respectively.

Using the EDS elemental quantification of elements, allow us to deliver a qualitative analysis of the worn and unworn surface of the balls after tribological tests. In the worn area are found elements not detected on the unworn surface. The new elements are S (up to 1 wt.%), P (0.5 wt.%), O (up to 3 wt.%), Zn (up to 3 wt.%), and Ca (up to 1 wt.%). These new elements occurring suggest the formation of a stable tribofilm at wear track. The above observation is sustained by the friction coefficient modification for the 0.075 wt.% TiO2 addition to oil compared with the pure oil. The friction coefficient decreases from 1.20 (pure oil) to 0.05 (oil with 0.075 wt.% TiO_2_) at 75 °C and from 1.8 (pure oil) to 0.4 (oil with 0.075 wt.% TiO_2_) at room temperature.

### Wear depth scar on the balls

The depth of the wear scar on the spheres was measured using an Alicona Inginite Focus G5 microscope. The surfaces were scanned with a microscope using 50 × magnification, and the light source was coaxial with the eyepiece (lenses) and supplemented with a light ring. Scanning was performed using Image Field mode with a vertical resolution between 0.003 and 0.032 μm and a horizontal resolution of 2.13 μm. The duration of a scan was between 1.5 and 3 min. The average scan height was 0.150 mm. This gives a Vertical Dynamic of 150/0.032 = 4687.5.

**Figure 10:**
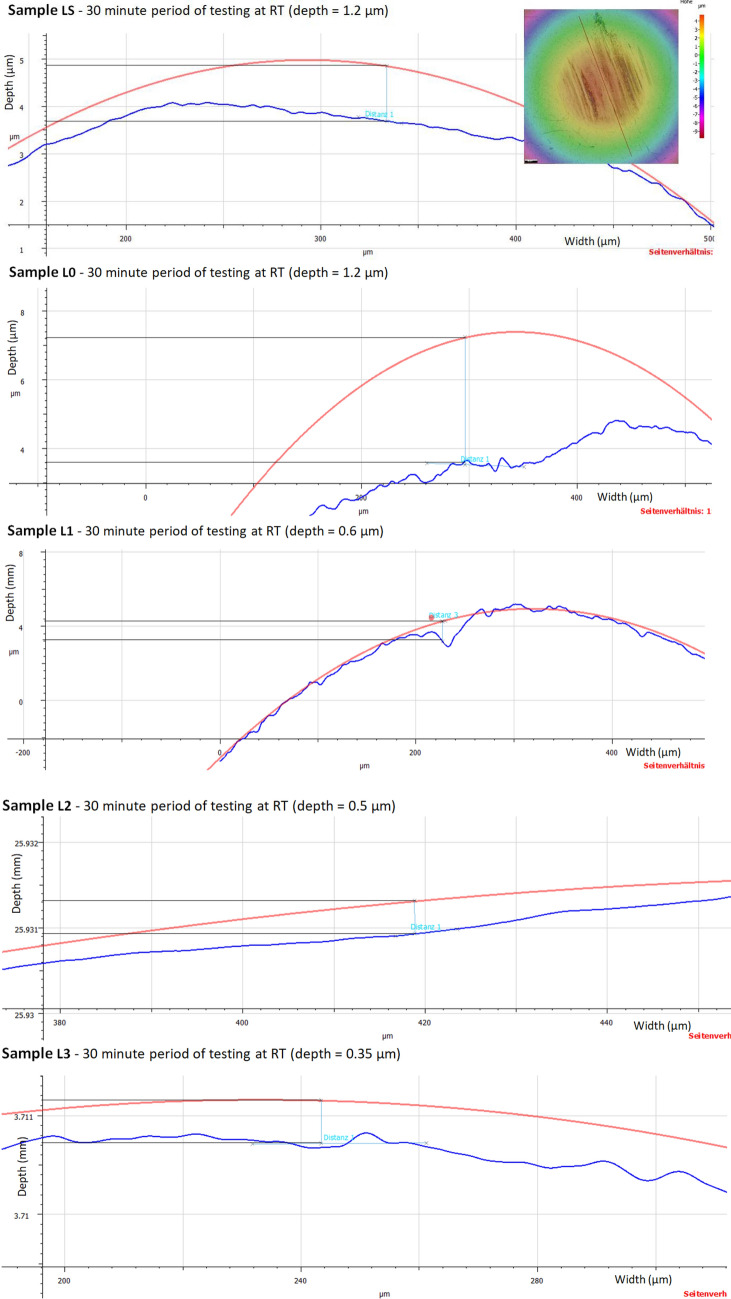
Wear depth scar (µm) of lower balls at room temperature (RT).

**Figure 11:**
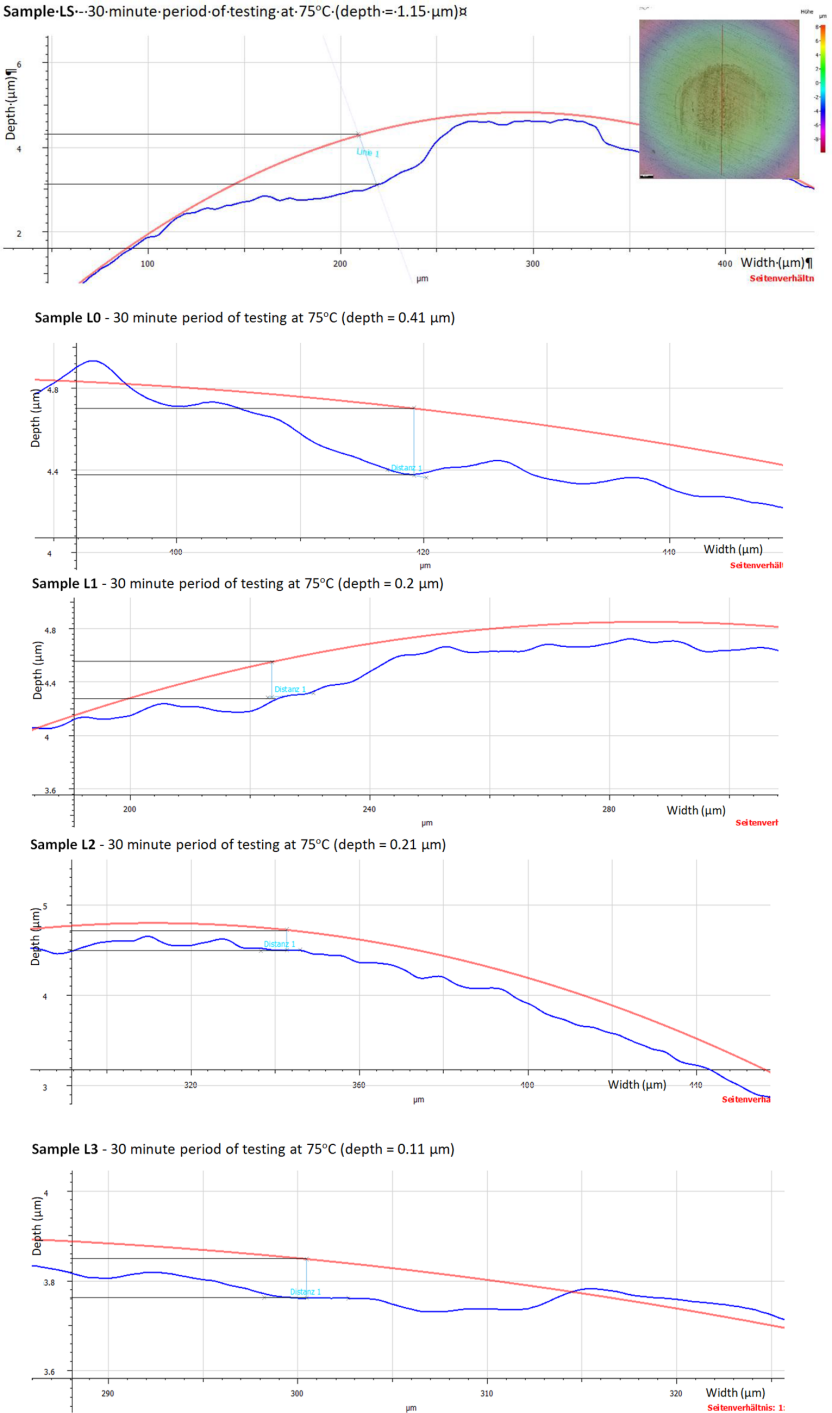
Wear depth scar (µm) of lower balls at 75 °C.

The evaluation of the wear depth was performed by measuring the distance from the ideal circle, constructed using a fixed 6.35 mm beam with the Measure Circle function. The traces of the intersection between the scanned surface and the plane in which the depth measurement was performed were used to orient and position the ideal circle. The depth of wear (difference between the ideal circle and the trace on the sphere) was measured using the measure height step function or maximum distance. The 2D profiles of the worn surfaces for different lubricant oils after the wear test are shown in Figs. 10 and 11.

Compared with pure oil, TiO_2_ nanoparticles added in the base oil had a significant improvement in the wear surface. After the wear test at RT, only small groves could be observed, and the wear mechanism was mainly formed by microplowing. Although the wear depth of the 0.01 wt.% sample was much smoother than that of the base oil, microplowing still existed, with a corresponding wear depth of 1.2 µm at RT and 0.41 µm at 75 °C. Moreover, the anti-wear property is improved with the amount of TiO_2_ nanoparticles added in the base oil. This indicates that TiO_2_ nanoparticles added in engine oil prevents the plowing wear that existed in the control sample.

**Figure 12:**
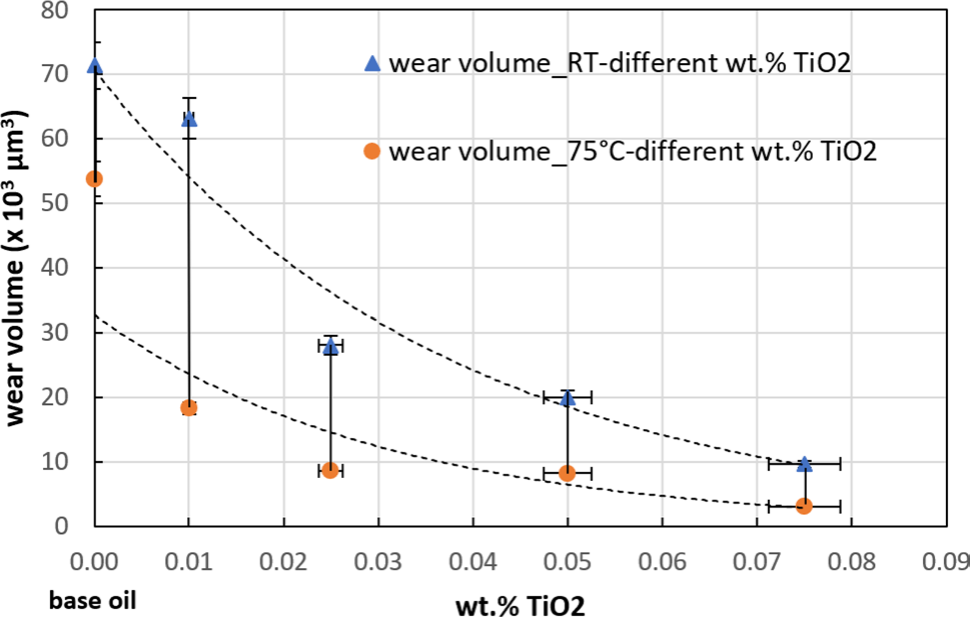
Ball wear volume for oils used in this work at different temperature.

The results of the wear traces analyzed in Figs. 10 and 11 were concatenated in Fig. 12. Figure 12 shows a chart of the amount of ball wear, as measured using the Alicona Inginite Focus G5 microscope. Very little wear protection was provided by the base oil, but the addition of nanoparticles to the base oil significantly reduced wear. In addition to the fact that it indicates the amount of wear obtained for the 4 types of tests, Fig. 12 also indicates the mode of wear behavior depending on the 2 temperature conditions for which the tests were performed (RT and 75 °C).

The wear rate of the lower balls at RT was somewhere 70 × 10^3^ μm^3^ for the base oil, gradually reaching up to 10 × 10^3^ μm^3^ for the concentration of 0.075 wt.% TiO_2_. At a temperature of 75 °C, the wear decreased to 33 × 10^3^ μm^3^ for the base oil, and then with the addition of wt.% TiO_2_ nanoparticles, it decreased to 5 × 10^3^ μm^3^. The measurements of the wear depths produced on the balls were accurate and repeatable with the help of the Alicona Inginite Focus G5 microscope.

In the current study, the authors tried to avoid overloading the tribocouple due to a high risk of layer deformation and change in the wear mechanism. Considering the wear rates of the balls studied by the authors (33–70 × 10^3^ μm^3^) at RT, it can be concluded that the wear of the balls is a few orders of magnitude larger for balls lubricated at 75 °C. This can be explained by the fact that the connecting rods are always in contact, and therefore, the phenomenon of continuous overheating occurs.

No transfer film was observed on the balls at a slip velocity of 0.80 ms^−1^. However, the tests were accompanied by vibrations and unwanted noise.

### Friction property of lubricating oils with TiO_2_ additives

**Figure 13:**
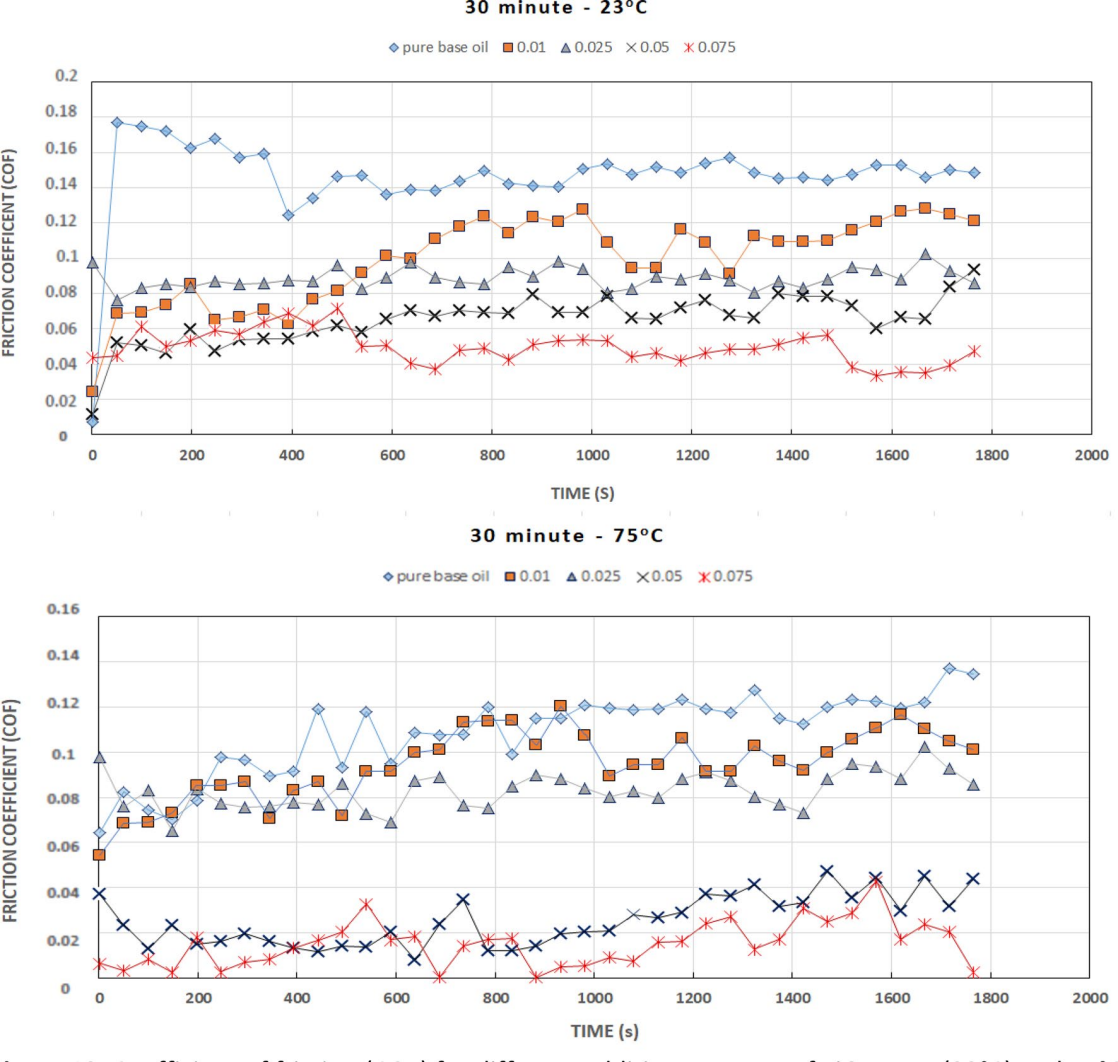
Coefficient of friction (COF) for different additive amounts of TiO_2_ at RT (23 °C) and 75 °C.

**Figure 14:**
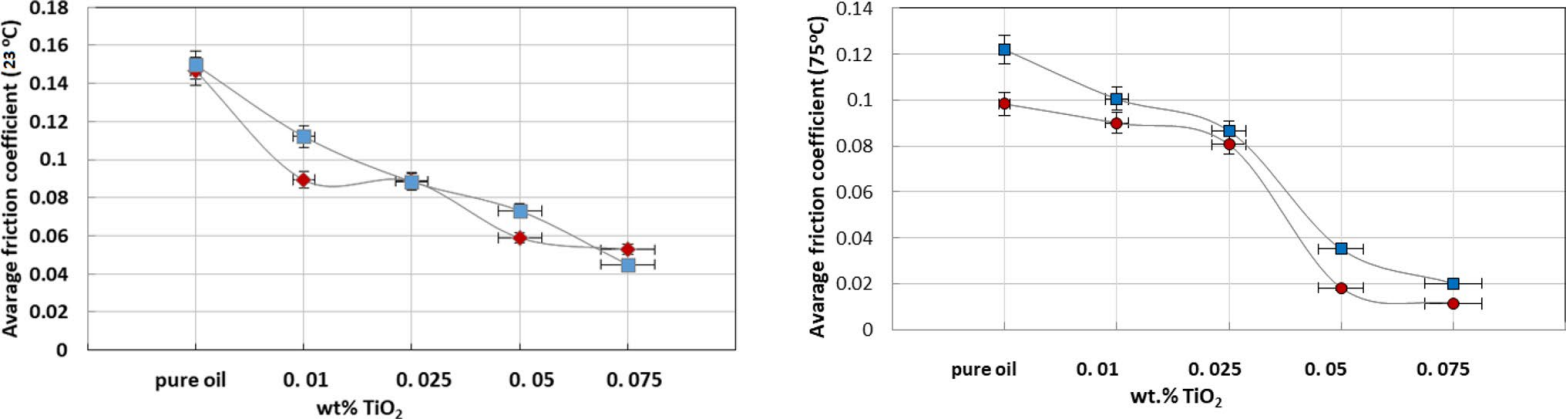
Average coefficient of friction (COF) for different additive amount of TiO_2_ at RT (23 °C) and 75 °C and COF in steady state after rubbing effect.

The tribological performance of engine oil (Motul 5100 4T10 W-30) with TiO_2_ additive loading as a lubricant additive is shown in Fig. 14. The coefficient of friction (COF) of engine oil with different additive TiO_2_ amounts was measured at an applied load of 10 N at the arm of the tribometer, 396 ± 4 N normal load applied on balls and rotation 1200 rpm, as presented in Fig. 13. Relative fluctuations in the friction response of base engine oil were observed in comparison to the additive response, and the COF was found to increase with time in the initial stage during the running period.

The coefficients of friction were remarkably reduced by the addition of TiO_2_ nanoparticles to the base lubricant. At a normal applied force of 396 N, the coefficient of friction of the nano lubricant was reduced by approximately 60% of the COF value for the base lubricant at RT and by approximately 80% at a temperature of 75 °C.

Clearly, the nano lubricant with more TiO_2_ nanoparticles had the best coefficient of friction (Fig. 13). These results indicate that the TiO_2_ nanoparticles decrease the ball-to-ball friction contact compared to the base lubricant.

The medium lowest COF of 0.01 was obtained by the oil sample with 0.075 wt.% TiO_2_ under a 75 °C lubricant temperature. Moreover, Fig. 13 shows the influence of particle concentration on the COF of oil suspensions, indicating that the average COF was influenced by the TiO_2_ concentration. The average COF obviously fell from 0.112 to 0.05 in the range of 0.01 wt.% to 0.075 wt.% TiO_2_, reflecting that the addition of nanoparticle lubricants straightened the sliding response when stabilizing additive amounts below 0.025 wt.% TiO_2_. However, the average COF showed a slightly decreasing trend from 0.010–0.088 in the range of 0.010 wt.% to 0.025 wt.% at RT. On the other hand, at 75 °C, the average COF obviously fell from 0.095 to 0.015 in the range of 0.01 wt.% to 0.075 wt.% TiO_2_. Furthermore, the COF tended to be stable after rubbing, and the corresponding average COF in the stable period for higher concentrations of TiO_2_ presented a lower antifriction property, as shown in Figs. 13 and 14. The relevant tribological mechanism at RT is due to TiO_2_ particles at higher concentrations accumulating in the inlet of the ball-on-ball contact area, which causes an insufficient supply of lubricant and starvation in the contact area.

The running-in period is of great significance to the regulation of tribological performance to a certain extent. Reducing the running-in period is beneficial to improving the antifriction property. The formation of a boundary lubrication film is the main reason for the stability of the friction coefficient. The coefficients of friction stabilized in the second part of the test time. The rubbing period of nano-oil with a 0.1 wt.% concentration lasted longer than that of the others with a time of 780 s. In addition, it is noteworthy that the rubbing time obviously decreased with increasing concentration. The 0.05 and 0.075 wt.% samples had the shortest rubbing time in terms of friction properties.

The friction coefficient was calculated according to IP-239 (EP and AW tests for lubricants) and is expressed as follows:

1 $$ \upmu = 0.22248 \cdot \frac{1000 \cdot T}{F} $$

where T is the frictional torque in kg·mm and F is the applied load in kg^12^. The frictional torque data were recorded by software, and the friction coefficient was calculated.

The frictional curve (Fig. 13) showed a slight increase in the value of COF at the start. However, after 25–30 min, the steady-state condition was attained, and curve remained approximatively stable thereafter. No abrupt behavior or sharp peak was observed in the steady-state region. The improved performance is attributed to the film formation capability of TiO_2_ nanoparticles.

### Flash temperature parameter

The flash temperature parameter is a unique number that gives us indications of the critical flash temperature above which the lubricant used will be out of use^16^.

The flash temperature parameter for operating conditions in the four-ball tribometer:

2 $$ \text{FTP} = \frac{\text{F}}{{\text{d}^{1,4} }}$$

A flash temperature parameter (FTP) was calculated for all the experimental conditions according to Eq. (2). In this equation, F is the normal load in kilograms and d is the mean wear scar diameter in millimeters at the load. A detailed explanation of the parameter is given by Lane^16,17^.

High values for the flash temperature parameter indicate that the lubricant shows good performance with a reduced possibility of lubricant breakdown^15^.

**Figure 15:**
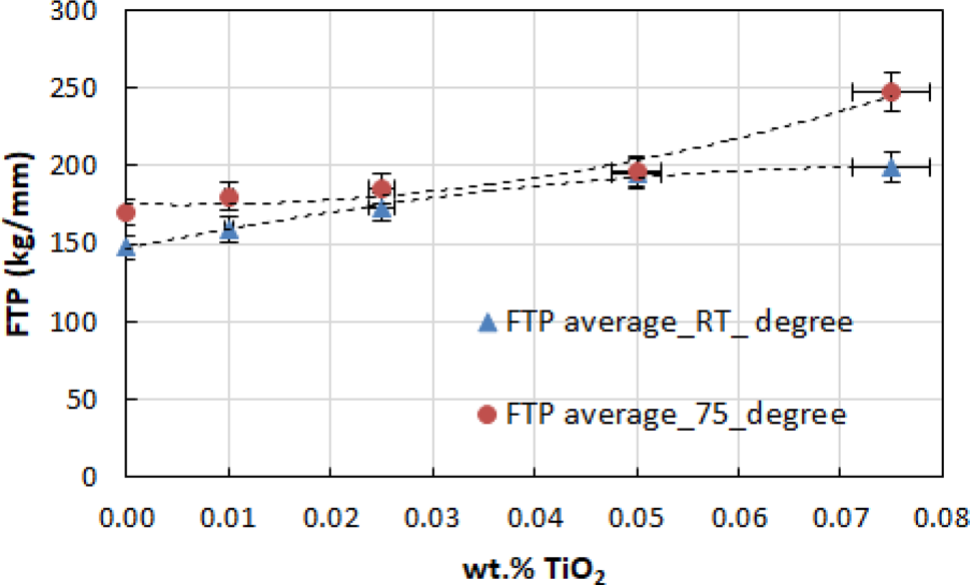
Plot of TiO_2_ as a lubricant additive vs. flash temperature parameter FTP for different temperature.

Figure 15 shows the plot of TiO_2_ percentage vs. flash temperature parameter (FTP) for different testing temperatures, more exactly room temperature RT and 75 °C. From the figure, the maximum and minimum FTPs were obtained from 0.075 wt.% contaminated lubricant and pure lubricant, respectively. The maximum FTP value means that good lubricating performance occurred, indicating a lower possibility of lubricant film breakdown. This phenomenon has also been observed by other researchers^11^. This seems to indicate that TiO_2_ nano additives are a potential anti-wear additive for lubricating oil. The 0.075 wt.% TiO_2_ in this investigation improved the lubricant performance based on the higher value of FTP observed compared with pure lubricant. The graphs also show the effect of temperature on the FTP of lubricants.

## Conclusions

For the tests performed on a four-ball wear machine with different percentages of TiO_2_-contaminated lubricant, the conclusions drawn are as follows.

The wear of the ball specimens decreases with the increase in wt.% TiO_2_ addition. Compared with pure lubricant, 0.075% TiO_2_ improves the lubricant performance based on the higher value of the flash temperature parameter (FTP).

The medium lowest COF of 0.01 was obtained by the oil sample with 0.075 wt.% TiO_2_ under a 75 °C lubricant temperature. The average COF obviously fell from 0.112 to 0.05 in the range of 0.01 wt.% to 0.075 wt.% TiO_2_, reflecting that the addition of nanoparticle lubricants straightened the sliding response when stabilizing additive amounts below 0.025 wt.% TiO_2_. On the other hand, at 75 °C, the average COF obviously fell from 0.095 to 0.015 in the range of 0.01 wt.% to 0.075 wt.% TiO_2_. The lowest COF was obtained for the 0.075 wt.% TiO_2_ oil sample, which dropped by approximately 60% compared to the pure base oil at RT and by approximately 80% at a temperature of 75 °C. Furthermore, the rubbing time obviously decreased with the increase in TiO_2_.

The main wear type of the worn surfaces lubricated by the engine base oil with TiO_2_ could be attributed to slight ploughing wear. Pure oil presented an obvious furrow and indentation, with a corresponding maximum depth of 1.2 µm. From physical observations on worn surfaces of specimens, it can be suggested that nano-oil with TiO_2_ (0.075 wt.%) acts as an anti-wear lubricant additive. Micrographs showed that wear scar surfaces in the 0.075% TiO_2_-contaminated lubricant tests appear to be much smoother, thus having less material transfer.

TiO_2_ plays a decisive role in the improvement of the tribological performance. Additionally, spherical TiO_2_ has a probable ball-bearing lubrication effect during the friction process. The optimum concentration of TIO_2_ nanoparticles further helps to form the film for a long time, due to its inherent characteristics. Thus, the experiment showed that the developed nano-fluids could serve potential engineering applications and help achieve the goals of sustainable development.

## Abbreviations

ASTM: American Society for Testing and Materials
AW: Antiwear
COF: Coefficient of friction
µ: Coefficient of friction
Eps: Extreme pressure properties
FTP: Flash temperature parameter
NPs: Nanoparticles
RT: Room temperature
SEM: Scanning electron microscopy
TBN: Total basicity number
WPs: Wear preventive properties
wt.%: Weight percent
E: Elastic modulus
HRC: Rockwell hardness
EDS: Energy dispersive X-ray spectroscopy
VC: Volume concentration

## References

[1] Dai W, Kheireddin B, Gao H, Liang H (2016). Roles of nanoparticles in oil lubrication. Tribol. Int..

[2] Binu KG, Shenoy BS, Rao DS, Pai R (2014). Static characteristics of a fluid film bearing with TiO_2_ based nanolubricant using the modified Krieger–Dougherty viscosity model and couple stress model. Tribol. Int..

[3] Ingole S, Charanpahari A, Kakade A, Umare SS, Bhatt DV, Menghani J (2013). Tribological behavior of nano TiO_2_ as anadditive in base oil. Wear.

[4] Demas NG, Erck RA, Lorenzo-Martin C, Ajayi OO, Fenske GR (2017). Experimental evaluation of oxide nanoparticles as friction and wear improvement additives in motor oil. J. Nanomater..

[5] Wu YY, Tsui WC, Liu TC (2007). Experimental analysis of tribological properties of lubricating oils with nanoparticle additives. Wear.

[6] Lee K, Hwang Y, Cheong S, Choi Y, Kwon L, Lee J, Kim SH (2009). Understanding the role of nanoparticles in nano-oil lubrication. Tribol. Lett..

[7] Jason YJJ, How HG, Teoh YH, Chuah HG (2020). A study on the tribological performance of nanolubricants. Processes.

[8] Ilie F, Covaliu C (2016). Tribological properties of the lubricant containing titanium dioxide nanoparticles as an additive. Lubricants.

[9] Shenoy B, Binu K, Pai R, Rao D, Pai RS (2012). Effect of nanoparticles additives on the performance of an externally adjustable fluid film bearing. Tribol. Int..

[10] Kao MJ, Lin CR (2009). Evaluating the role of spherical titanium oxide nanoparticles in reducing friction between two pieces of cast iron. J. Alloys Compd..

[11] Arumugam S, Sriram G (2013). Preliminary study of nano and micro scale TiO_2_ additives on tribological behavior of chemically modified rapeseed oil. Tribol. Trans..

[12] Wang Y, Lei T, Guo L, Jiang B (2006). Fretting wear behaviour of microarcoxidation coatings formed on titanium alloy against steel in unlubrication and oil lubrication. Appl. Surf. Sci..

[13] Ingolea S, Charanpahari A, Kakade A, Umare SS, Bhatt DV, Menghani J (2013). Tribological behavior of nano TiO_2_ as an additive in base oil. Wear.

[14] Gupta G, UlHaq MI, Raina A (2021). Rheological and tribological behavior of sunflower oil: Effect of chemical modification and tungsten disulfide nanoparticles. J. Bio Tribo Corros..

[15] Raina A, Ul Haq MI, Anand A, Mohan S, Kumar R, Jayalakshmi S, Arvind Singh R (2021). Nanodiamond particles as secondary additive for polyalphaolefin oil lubrication of steel-aluminium contact. Nanomaterials.

[16] Anand R, Raina A, Ul Haq MI, Mir MJ, Gulzar O, Wani MF (2021). Synergism of TiO_2_ and graphene as nano-additives in bio-based cutting fluid—An experimental investigation. Tribol. Trans..

[17] Kerni L, Raina A, Ul Haq MI (2019). Friction and wear performance of olive oil containing nanoparticles in boundary and mixed lubrication regimes. Wear.

[18] Abdullah MIHC, Abdollah MFB, Amiruddin H, Tamaldin N, Nuri NRM (2016). The potential of hBN nanoparticles as friction modifier and antiwear additive in engine oil. Mech. Ind..

[19] Habibullaha M, Masjukia HH, Kalama MA, Ashrafula AM, Habibb MA, Mobaraka HM (2014). Effect of biolubricant on tribological characteristics of steel. Procedia Eng..

[20] Ali MKA, Xianjun H, Turkson RF, Peng Z, Chen X (2016). Enhancing the thermophysical properties and tribological behaviour of engine oils using nanolubricant additives. R. Soc. Chem. Adv..

[21] Masjuki, H. H., Saifullah, M. G., Husnawan, M., Faizul, M. S. & Shaaban, M. G. Flash temperature parameter number prediction model by design of tribological experiments for basestock mineral oil containing palm olein and aminephosphate additives. Proceedings of the World Tribology Congress III, 451–452. 10.1115/WTC2005-63193 (2005).

[22] Ali MKA, Xianjun H, Mai L, Qingping C, Turkson RF, Bicheng C (2016). Improving the tribological characteristics of piston ring assembly in automotive engines using Al2O3 and TiO_2_ nanomaterials as nano-lubricant additives. Tribol. Int..

[23] Yang L, Du K, Zhang XS, Cheng B (2011). Preparation and stability of Al_2_O_3_ nano-particle suspension of ammonia-water solution. Appl. Therm. Eng..

[24] Molea, A., Barabas, I. & Suciu, R. Influence of TiO_2_ nano-particles content on physicochemical and tribological properties of lubricant oil. Proceeding of the 4th International Congress of Automotive and Transport Engineering (AMMA 2018), Book Series: Proceedings in Automotive Engineering, 190–196. 10.1007/978-3-319-94409-8_23 (2019).

